# Editorial Notes

**Published:** 1910-06

**Authors:** 


### Editorial Notes.

---

**Honorary Medal, R.C.S.**
(*Vide* p. 190.)

Those of us who appreciate the advantages of a large medical library know how indispensable it is to have bibliographical facilities of access to its resources. It would be difficult to name any one who has more benefited his fellows in this respect than Dr. Robert Fletcher, whose work in connection with the *Index Medicus* and *The Index-Catalogue of the Library of the Surgeon-General's Office* at Washington is beyond all praise. As Dr. Fletcher is a Bristolian by birth and was a student at our Infirmary, we should very heartily join in the congratulations accorded to him upon the receipt of the Honorary Medal which the Royal College of Surgeons of England has recently by a graceful act bestowed upon him in recognition of his labours. Dr. Fletcher has shown from time to time a keen interest in the medical work of his old neighbourhood.

**Proprietary, Patent and Secret Remedies.**

The recent discussion of this subject by the Therapeutical and Pharmacological Section of the Royal Society of Medicine (vide *Proceedings*, March and April, 1910) will have done much good in directing the attention of prescribers to the great abuse of doubtful remedies outside the official pharmacopœia. Dr. Dixon remarks that, after a long period of something like chaos in the drug world, " now, when the dawn of a new age is thick upon us, and empiricism is being replaced by rationalism, it is hardly a matter for wonder that there are those of us who would substitute the laboratory for the bedside in all pertaining to therapeutics; others who would rely entirely on the bedside; and a third group of ardent and enthusiastic believers in all new products and methods of treatment of diseases. Some regard baths, mineral waters, electricity, or radium as the panacea for all diseases, and others put their trust in the lactic-acid bacillus, calcium, or whatever other drug is in vogue at the time." In spite of the large number of new drugs which the chemical manufacturers and the enterprising journalists and advertisers are constantly pouring upon us, there is a growing tendency to trust to all other methods of treatment rather than drugs, which may be left to be distributed by the advertising druggist and taken on his advice.

Now the proprietary element has been the great source of our difficulties. Many of these combinations are admitted to be an improvement on the crude mixtures which are often prescribed in a haphazard way, the result of the combination being untested except by the unfortunate patient on whom it is bestowed, whereas the manufacturer, who has expended much time and care in evolving his drug or his combination of drugs, commonly produces a something which commends itself empirically to the patient if not to the doctor. By giving a distinctive name to his product and by showering samples broadcast amongst those whose assistance is needful, he persuades the medical profession to prescribe his substance under its fancy name which ensures his proprietary claim. Before long some other manufacturer wishes to share in the profits, and he issues a precisely similar drug under a new name, and claims that his preparation is superior. Hence the chaos which the much-abused *British Pharmaceutical Codex,* published in 1907, did its best to remove. It gave the composition of certain substances which had been known under trade names, and immensely simplified the work of the druggist in dispensing. It was intended to be a compendium of medicines for which there might be any demand, and it enabled the dispenser to make for himself a large number of combinations which had previously been proprietary. It is now stated that "the *Codex* has confused prescribers, was pernicious to the public, and it violated the first principles of literary copyright." We think that this attack on the *Codex* was much to be deprecated, but the critics admit that its errors were due more to want of thought than to want of knowledge. It will be impossible for the general physician to avoid the use of proprietary articles entirely, but much could be done if the *Codex* came into more general use. It seems to be impossible to prohibit from the general prescribers' use all drugs and combinations not included in the *Pharmacopœia.* This being granted, the *Codex* should be a friendly auxiliary, and it cannot fail to be of great use both to the dispenser and the prescriber.

Meanwhile Dr. Dixon reminds us " that the cure for these evils lies in our own hands. We should never prescribe a secret remedy ; if it has any beneficial action it must be due to one or more well-known drugs, which can be easily ascertained. I think we ought not to prescribe a compound medicine of a proprietary nature, especially such things as compressed tablets, in the original packages : it is a direct means of encouraging self-drugging. We ought not to prescribe a drug with a registered fancy name unless it is held by Letters Patent, but refer to its synonym, and prescribe it by its chemical name, unless distinct reasons exist for prescribing the original product."

These are suggestions which we should do well to remember, and so simplify the work of the dispensing chemist, who must often be driven to distraction in endeavouring to comply with the wishes of the prescriber.

**L'Enseignement Médico-mutuel International.**

It will be remembered that last summer many of us had the gratification of welcoming a party of the International Postgraduate Society, when they included Bristol in their annual tour. Shortly after the time of that visit, Dr. Etienne Bazot, the President of the Association and Secretary-General of the French National Committee, was very seriously ill. We now have the pleasure to congratulate him on his recovery, and trust that he may long be spared to carry on the work of this prosperous Society, of which he was the founder. We have received the first three numbers of the *Journal* of the Society for this year, and hope to continue to receive the monthly issue in exchange for our own *Journal.* The March number contains the first chapter of the Compte-Rendu of the Voyage d'Etudes of 1909 to Angleterre, Ecosse,

Irlande. An interesting account of the reception of the Society by the municipality of Boulogne gives us the valuable hint that champagne is an excellent preventive of sea-sickness.

**Edridge Green's Lantern Test.**

At a recent meeting of the Society Mr. Cyril H. Walker called attention to the recent improvements in tests for colour vision. It has, of course, for a long time been notorious that the use of Holmgren's wools as the accepted test for colour vision is in several respects unsatisfactory. Used as originally directed, they do not in the least resemble or reproduce the conditions at sea or on the railway where accurate colour vision for small objects (or objects subtending a small visual angle) is most needed. Not that Holmgren's wools are of no value, or have been entirely superseded. They are convenient for rapidly detecting gross colour defects, and also for ascertaining that candidates have a correct knowledge of colour nomenclature, such as is required for the proper recognition of flags and signals. One respect in which the wool test is a failure lies in the fact that the candidate is guided to a correct selection by being able to compare any doubtful colours with the rest of the colours in the bundle. For example, a candidate with "shortening" (*i.e.* imperfect appreciation of) the red end of the spectrum might group the red wools together correctly. He would see that they did not agree with the hue of any others of the wools before him, and would infer that they were what others called "reds" without his being able to appreciate the red colour himself.

For scientific purposes the spectrum is undoubtedly the most accurate test of colour vision, since it is possible to record the exact wave-length of any colour which is imperfectly seen. Most of the spectrum tests involve costly and elaborate apparatus, but Dr. Maitland Ramsay (Ramsay's test) has recently devised an instrument which is in many respects ideal. Essentially it consists of a tube about a foot long with an eye piece at one end and a 16 c.p. electric lamp at the other. In the middle is a diffraction grating, suitably illuminated by two adjustable slits near the lamp, by which two similar spectra are produced. By means of movable shutters any portion (for example, a narrow strip of green) can be isolated in the lower spectrum. The patient is then told to move similar shutters by means of a screw with a milled head until an exactly similar patch of green has been isolated in the upper spectrum. Any defect is recorded on two graduated dials on the top of the tube. The objections to this instrument are, first, that the conditions of the test are quite unlike those which exist in actual service; secondly, the use of the instrument requires considerable intelligence on the part of the subject who is being tested, and stupidity might rank as a colour defect; further, it is not very easy with it to convince a third party of the candidate's faulty vision.

To Dr. Edridge Green belongs the credit of having invented what is after all by far the most practical, if not the most scientific test for colour vision. More than twenty years ago he pointed out the necessity of a better test than was afforded by Holmgren's wools. Without discussing his theories as to colour vision, it may be said that he assumes that the ordinary eye is "hexachromic," *i.e.* can distinguish red, orange, yellow, green, blue and violet as being six different kinds of colour. His first step, therefore, was to select most carefully glass of suitable colours so as to transmit rays of light from certain known portions of the spectrum, chosen of course with a view to detecting errors of colour vision. For example, he uses two "reds," one of which transmits some green rays as well. Those who have a shortened red end of the spectrum will very likely not appreciate the red and call the colour green. The latest model of his apparatus consists of an opaque disc about 8 inches in diameter with a circular aperture near its periphery, which can be varied from about $\frac{1}{8}$ inch to $\frac{3}{4}$ inch diameter by means of a rotating diaphragm. By thus diminishing the size of the aperture the effect of a distant signal can be imitated. Behind this aperture three discs, bearing the selected test glasses, can be rotated so as to mix the colours as required (three greens, two greens and a yellow, one green, one yellow and one blue, etc.). There is, in addition, a fourth disc provided with ground and neutral tint glasses, by means of which the effect of atmospheric conditions—rain, fog, smoke, etc.—can be imitated. The denser of these neutral tint glasses diminish the rays from the most luminous parts of the spectrum, so that a white light begins to assume a smoky orange tint. A candidate who was red-green blind might interpret this as a red or green signal. The apparatus is illuminated by an electric lamp, the light of which is reflected through the glass at the sight hole to the eye of the patient to be examined, who is seated a yard or two off. It must be confessed that a more constant source of light is desirable.

The candidate to be examined is first tested with Holmgren's wools in order to ascertain that he can name colours correctly, and to exclude him at once if he is hopelessly defective. He is then placed before the instrument and asked to name the colours as the examiner rotates the discs. The examiner can see and knows by the colours used if the candidate makes any mistakes, and the correctness of the answers can be judged by three or four witnesses standing near the candidate.

**Dr. Robert Koch.**

FOR the following note we are indebted to the courtesy of Dr. Barclay J. Baron, who writes:—

"On Saturday, May 28th, our profession lost one of its most distinguished members by the death, at Baden-Baden, of Robert Koch.

"Graduating at Göttingen in 1866, he had crowded such an amount of successful scientific research into forty-four years of professional life as has rarely been equalled, and never excelled.

"From the time when he was appointed Medical Officer of Health at Wollstein he was ever on the hunt for a microbe as the specific cause of disease. In 1876 came his first great find—if one may so call it—when he discovered the microbe of anthrax, and thus laid the foundation of his imperishable fame. It was then that he formulated the four postulates that have ever since been regarded as essential by all workers in bacteriological research.

"In 1882, as all the world knows, he discovered the bacillus of tuberculosis, which had long been sought for by bacteriologists. This discovery, with all the possibilities of efficient help to countless multitudes of stricken people and animals, at once placed Koch in the very forefront of medical great ones. Henceforth we classed him with Lister and Pasteur.

"With endless energy he investigated tick fever in Texan cattle, and rinderpest in South Africa, and in 1883 he definitely placed the comma bacillus as the cause of cholera. In this connection we do not forget the remarkable work of Budd, Brittan and Swayne long years before.

"In 1897 we find Koch testing Kitasato's discovery of the germ of bubonic plague, and confirming, by his researches, Manson's theory of the spread of the disease by migratory rats. One of his last inquiries was into the origin of "sleeping sickness," which he attributed to inoculation by the fly *glossina palpalis*, which obtains the germ from the crocodile.

"Following Jenner, it was natural that Koch should try and find remedies by "vaccination," and the enormous sensation caused by the announcement in 1890 that he had found a cure for tuberculosis is still fresh in our memory.

"In order to see for ourselves what was being done in Berlin, where the use of tuberculin was being tried on a huge scale, Dr. Markham Skerritt and I went to that city, armed with introductions which gave us the run of the hospitals and clinics. We saw crowds of patients exhibiting the reactions due to the injections, and we were shown healing and healed ulcerations that were said to have taken on healthy action after its use. There was such an extraordinary amount of enthusiasm on the part of all those who saw the patients—professors, hospital physicians and surgeons, and visiting medical men from all parts of the world—that it was almost impossible to take guarded views of results, although everyone knew that in tuberculosis, of all diseases, caution was most necessary. Berlin was clearly not the place—it was too near to the wonderful man Koch—to cultivate this most necessary caution. As we know, it was reserved for Virchow to quietly undermine the extravagant claims that were put forth—but we must in justice say—not by the great investigator and discoverer himself, whose hand had been, unfortunately, forced by those in high places.

"Dr. Skerritt and I visited him, and found his flat besieged by admirers, and (I fear I must add) beggars for a phial of the new fluid. He granted us a few minutes of pleasant chat, but refused to talk of results of the new treatment, and so far as obtaining any tuberculin—he sent us empty away. He struck us as a perfect type of what a great investigator should be—calm, modest, and non-committal.

"Vaccine therapy has made great strides since those days, and the names of Behring, Wright and others come to mind at once as great living authorities; but we shall all ascribe to Robert Koch the credit of having been the pioneer in this form of treatment, the benefits of which no man can estimate."

**Elizabeth Blackwell, M.D.**

THE first woman to establish the right of her sex to enter in modern times the medical profession has recently died at the advanced age of 90 years.

Women held a well recognised position as practitioners of medicine in the Middle Ages, and several of the *matronæ*, or *mulieres Salernitanæ*, attained renown at the school of Salernum. The best known is Trotula de Ruggiero, who wrote *De Mulierum Passionibus*. During the succeeding centuries monasticism rose and laid hold on all the seats of learning in Western Europe, with the result that for nearly six centuries the professions were closed to women. About 1820 Elizabeth Blackwell was born in Bristol. When she was eleven years old her father, Samuel Blackwell, emigrated with his wife and family to the United States. For six years he struggled against adversity, then died, leaving his family entirely unprovided for. Elizabeth, who was the third of nine children, with her sisters, set up a school in New York State. In the early forties she determined to enter the medical profession, and after various rebuffs succeeding in gaining admission to the University of Geneva, U.S.A., where she eventually graduated as M.D.

In 1849 Elizabeth Blackwell returned to England, and by the good offices of Sir James Paget was admitted to the wards of St. Bartholomew's Hospital. Of this event Lady Paget wrote (October 17th, 1850): "Well, we have our 'Lady Doctor' here at last, and she has actually attended two of James' lectures, taking her seat with perfect composure. The young men have behaved extremely well, and she really appears likely to go on her way unmolested. She breakfasted here one morning with several of our students, and last evening we had a few medical friends to dinner, when she joined us in the evening. Her manners are quiet, and it is evident her motives for the pursuit of so strange a vocation are pure and good. So let us hope she will become useful in her generation."

She lived long enough to see the barriers which she had forced apart completely broken down, until all the universities in this country, with the exception of Oxford and Cambridge, have thrown open their classes and degrees to women students. "As Cabot opened up a new world in the material sense, so Elizabeth Blackwell (to quote the *Western Daily Press*), setting out also from the city of Bristol, revealed and conquered a new world for her own sex, and broke the first links of the chain which so long had bound women to conventions in the matter of medical science." In the long list of women who have lent lustre to the records of Bristol, the name of Elizabeth Blackwell will hold an honourable place.

